# BLINKER: Automated Extraction of Ocular Indices from EEG Enabling Large-Scale Analysis

**DOI:** 10.3389/fnins.2017.00012

**Published:** 2017-02-03

**Authors:** Kelly Kleifges, Nima Bigdely-Shamlo, Scott E. Kerick, Kay A. Robbins

**Affiliations:** ^1^Department of Computer Science, University of Texas at San AntonioSan Antonio, TX, USA; ^2^Qusp Labs, QuspSan Diego, CA, USA; ^3^US Army Research LaboratoryAberdeen, MD, USA

**Keywords:** eye blinks, blink duration, EEG, artifact, EEGLAB, machine learning, big data, human behavior

## Abstract

Electroencephalography (EEG) offers a platform for studying the relationships between behavioral measures, such as blink rate and duration, with neural correlates of fatigue and attention, such as theta and alpha band power. Further, the existence of EEG studies covering a variety of subjects and tasks provides opportunities for the community to better characterize variability of these measures across tasks and subjects. We have implemented an automated pipeline (BLINKER) for extracting ocular indices such as blink rate, blink duration, and blink velocity-amplitude ratios from EEG channels, EOG channels, and/or independent components (ICs). To illustrate the use of our approach, we have applied the pipeline to a large corpus of EEG data (comprising more than 2000 datasets acquired at eight different laboratories) in order to characterize variability of certain ocular indicators across subjects. We also investigate dependence of ocular indices on task in a shooter study. We have implemented our algorithms in a freely available MATLAB toolbox called BLINKER. The toolbox, which is easy to use and can be applied to collections of data without user intervention, can automatically discover which channels or ICs capture blinks. The tools extract blinks, calculate common ocular indices, generate a report for each dataset, dump labeled images of the individual blinks, and provide summary statistics across collections. Users can run BLINKER as a script or as a plugin for EEGLAB. The toolbox is available at https://github.com/VisLab/EEG-Blinks. User documentation and examples appear at http://vislab.github.io/EEG-Blinks/.

## Introduction

Contamination of electroencephalography (EEG) by eye and muscle activity is an ongoing challenge, and many techniques exist for the removal of these artifacts (Jung et al., 2000; Delorme et al., 2007; Nolan et al., 2010; Mognon et al., 2011; Winkler et al., 2011). On a parallel track, human performance characterization uses ocular indices to characterize fatigue and other changes in subject state (Schuri and von Cramon, 1981; Recarte et al., 2008; Benedetto et al., 2011; Wilkinson et al., 2013; McIntire et al., 2014; Marquart et al., 2015). Eye movements also integrally relate to perception and attention. Although direct measurement of eye activity is desirable, it is also possible to extract some types of ocular indices directly from EEG without additional experimental considerations. As large collections of EEG become available, these approaches enable the study of the distributions of ocular indices across many experimental conditions, diverse subject pools, and various disease conditions.

This work investigates the identification of blinks and the extraction of standard ocular indices related to eye blinks from EEG and/or electrooculography (EOG) in an automated fashion. The ocular indices that can be easily extracted from EEG include blink rate (*BR*), blink duration (*BD*), blink amplitude deviation ratio (*BAR*), positive amplitude velocity ratio (*pAVR*), negative amplitude velocity ratio (*nAVR*), percent time closed (*%TEC*), as well as standard deviations, rates of change and ratios of these measures. Continuing themes in research on these ocular indices are significant individual differences in these indicators across subjects and consistent relationships among subjects to levels of perceived sleepiness and to time-of-day (Ingre et al., 2006; Sandberg et al., 2011).

Blink duration, usually measured in seconds or milliseconds, typically ranges from 0.1 s to 0.5 s, but can go as high as 2 or 3 s as subjects start to fall asleep. Ftouni et al. (2013) found that blink duration began to increase dramatically after subjects had been awake for 18 h, reaching a peak at 28 h and then falling to a local minimum at around 34 h of continuous wake time. Blink duration also showed a strong circadian relationship, rising sharply after the subject's core body temperature reached a minimum. Ftouni et al. demonstrated that perceived sleepiness as measured by the Karolinska Sleepiness Scale (KSS) (Akerstedt and Gillberg, 1990) follows the same cyclic behavior as mean performance and mean reaction time lapses in a PVT (psychomotor vigilance task). Ftouni et al. also demonstrated similar cyclic patterns for *pAVR, nAVR*, and *%TEC*.

In addition to circadian and time-awake influences, blink duration and KSS show a large variability among subjects. Ingre et al. (2006) characterized significant differences among subjects in baseline blink duration, KSS, and lane deviation values in a driving simulation experiment. However, most subjects had larger values over baseline for all three measures when the same experiment was conducted at night. Sandberg et al. (2011) reported similar results for real-world driving. Using linear mixed-effect regression, Sandberg et al. showed that several measures, including blink duration varied linearly with KSS and with lateral lane position. However, the intercepts for these linear models varied considerably across test subjects. In another real-world driving experiment, Anund et al. (2013) showed that observer-rated sleepiness of driver behavior was also moderately correlated with blink duration.

Blink rate depends on many factors related to general visual function, eye physiology (such as the corneal tear film), facial movement, cognition, and level of arousal (Karson, 1988). Karson et al. (1990) showed that blink rates were higher than controls in individuals with schizophrenia and lower than controls in patients with Parkinson's disease (Karson et al., 1984). Research in both humans and primates indicates that blink rates have a significant social component in addition to other factors (Tada et al., 2013). In monkeys, Kleven and Koek (1996) showed significant increases in blink rate with the applications of high-efficiency dopamine agonists. Doughty (2014) showed that blink rate can also be influenced by other factors such as eye position and visual glare.

McIntire et al. (2014) showed that blink rate and blink duration increased as performance in vigilance tasks declined. They also showed that right cerebral blood flow velocity declined as performance declined and suggested that eye blink measures could be used as an operational indicator of arousal levels. Colzato et al. (2008) used daytime blink rate as a functional marker for attentional blinks. Attentional blinks refer to masking of a second visual target that appears within 100–500 ms of a first target. They show a negative correlation between blink rate and the attentional blink effect. In earlier work Colzato et al. (2007) showed that larger attentional blink effects are associated with a smaller operational span of working memory.

The amplitude-velocity ratio introduced by Johns (2003) relates directly to drowsiness. The *pAVR* is the ratio of the maximum signal amplitude to the maximum eye-closing signal velocity for the blink, while the *nAVR* is the ratio of the maximum signal amplitude to the maximum eye-opening signal velocity for the blink. Both ratios are in units of time, independent of the units of the amplitude. BLINKER converts the time units to *centiseconds* for comparison with values reported in the literature. Johns found a mean *pAVR* of about 4 centiseconds for alert subjects, with a normal range from 2.5 to 5.7 centiseconds. In sleep-deprived subjects, the mean *pAV*R rose to around 7 centiseconds. Johns observed that this mean was a result of periods of normal *pAVR* interspersed with intervals of very long blinks and high *pAVR*s. Johns postulates that some of the observed drowsiness lapses involved central neural inhibition of vision.

Putilov and Donskaya (2013) developed an EEG-based test called the Karolinska drowsiness test (KDT), which uses several EEG spectral measures including the difference between alpha and theta band powers and the value of the second principal component of the EEG spectrum (Makeig and Jung, 1995) during a 5 min eyes closed test. They showed that KDT correlated well with subjective measures of drowsiness such as the KSS. Ftouni et al. (2013) showed that performance lapses and ocular measures including blink duration, *pAVR, nAVR*, and *%TEC* also correlate well with EEG power changes in particular frequency bands including delta-theta (0.5–5.5 Hz), theta-alpha (5.0–9.0 Hz), and beta (13.0–20.0 Hz) as well as with KSS.

Although many studies have established a relationship between ocular indices and subject states such as fatigue, drowsiness, attention, and arousal, most studies have been relatively small. In addition, there appears to be considerable variability among subjects and even within the same subject based on initial state. In this work, we examine the ocular indices computed directly from EEG, focusing on indices related to blinks. We examine different ways of computing these indices and quantify the way these values differ over signal selection, method of computing the ocular index, subject, and type of task for a large corpus of EEG. In order to accomplish this task, we have developed a collection of tools and an automated pipeline for extracting ocular indices from EEG data. We have made the pipeline freely available as a MATLAB toolbox called BLINKER (https://github.com/VisLab/EEG-Blinks) in hopes that more researchers will report the values of ocular indices from their EEG studies.

The remainder of the paper is organized as follows. Section Methods and materials describes the steps and methods used to extract blinks from EEG data, as well as the datasets we used to validate the results. Section Results presents the results of running the BLINKER toolbox on a large and varied collection of EEG data. Section The BLINKER software briefly introduces the toolbox, referring readers to the more detailed online documentation and downloads. Section Visual verification of BLINKER output describes our manual video verification process and some interesting results we obtained when comparing subject behavior captured in video with blink signals captured by EEG. Section Discussion offers some concluding remarks and discusses limitations of the method.

## Methods and materials

### Datasets used in this study

Table 1 summarizes the EEG collections used in this study. The ARL-BCIT data collection consists of 669 datasets from 210 unique subjects performing simulated driving, rapid serial visual presentation (RSVP), and guard duty tasks. The NCTU-LK collection consists of 40 subjects in 80 sessions performing a lane-keeping task with and without a motion platform. NCTU-LK, which uses a 33-channel Neuroscan, was contributed from an extensive archive of driving studies performed at the National Chiao Tung University by C-T Lin and his collaborators (Chuang et al., 2012, 2014a,b). The ARL-Shoot study, is a task-load shooting study performed by Kerick and collaborators at the Army Research Laboratory using a 40-channel Neuroscan headset (Kerick et al., 2007). The collection consisted of 9 sessions for each of 14 subjects, resulting in 126 datasets. We also analyzed the publicly available BCI-2000 data collection, a 109-subject motor imagery data collection contributed by Shalk and colleagues to Physionet (Goldberger et al., 2000; Schalk et al., 2004). This collection of 1308 datasets contains 12 sessions for each of 109 subjects using a 64-channel BCI2000 headset. The datasets in the ARL-Shoot and BCI-2000 collections are relatively short in duration, and each subject performed the multiple tasks in a single session. For some analyses, as noted below, we combine the data for each subject in these collections into a single dataset for analysis.

**Table 1:** **Summary of the data collections used for this study: 2183 datasets from 370 unique subjects**.

**Collection**	**Headsets**	**Datasets**	**Unique subjects**	**Length in hours**	**EOG**	**Tasks**
ARL-BCIT	Biosemi (64 or 256 Ch)	669	210	426.4	Yes	Driving with perturb, RSVP, guard duty
ARL-Shoot	Neuroscan (40 Ch)	126	14	14.5	Yes	Shooting, arithmetic, dual shooting/arithmetic
NCTU-LK	Neuroscan (32 Ch)	80	37	104.5	No	Driving with perturb motion/motionless
BCI-2000	BCI2000 (64 Ch)	1308	109	44.8	No	Motor imagery (4 versions)

### Data preprocessing

The results reported in this paper use unprocessed EEG channel and EOG signals. However, we also tested our algorithms on datasets that had been robust average referenced using the PREP pipeline (Bigdely-Shamlo et al., 2015) and on independent components extracted after PREP. PREP removes line noise, interpolates bad channels, and performs a robust average reference. We used the Infomax (Bell and Sejnowski, 1995) independent component algorithm (ICA) as implemented in the EEGLAB runica function to compute independent components for the IC versions of blink extraction. Infomax does not always converge for noisy datasets. NCTU-LK and ARL-Shoot were mastoid-referenced at acquisition time, and the “raw” data in these collections refers to mastoid-referenced data. The “raw” data for ARL-BCIT used the default Biosemi CMS/DRL reference, while the acquisition reference strategy for BCI-2000 was not known.

### Extracting blinks from a time series

In clean datasets, candidate time series corresponding to front channels, an upper vertical EOG channel, or frontally focused ICs activations contain good representations of blink behavior. However, in a given dataset some of these signals may be missing or corrupted. Thus, an automated process should provide options for extracting blinks from multiple sources and possibly combining the information. In this section, we refer to the time series (EEG channel, EOG channel, or IC activation) from which we are attempting to extract blinks as the “signal.” The large variety and variability of eye and eyelid movements among subjects and tasks makes it difficult to capture all blinks in every data set using an automated technique. Hence, BLINKER uses several stages of processing and provides indicators of when the algorithm is working well and when the results may not be accurate.

**Figure 1:**
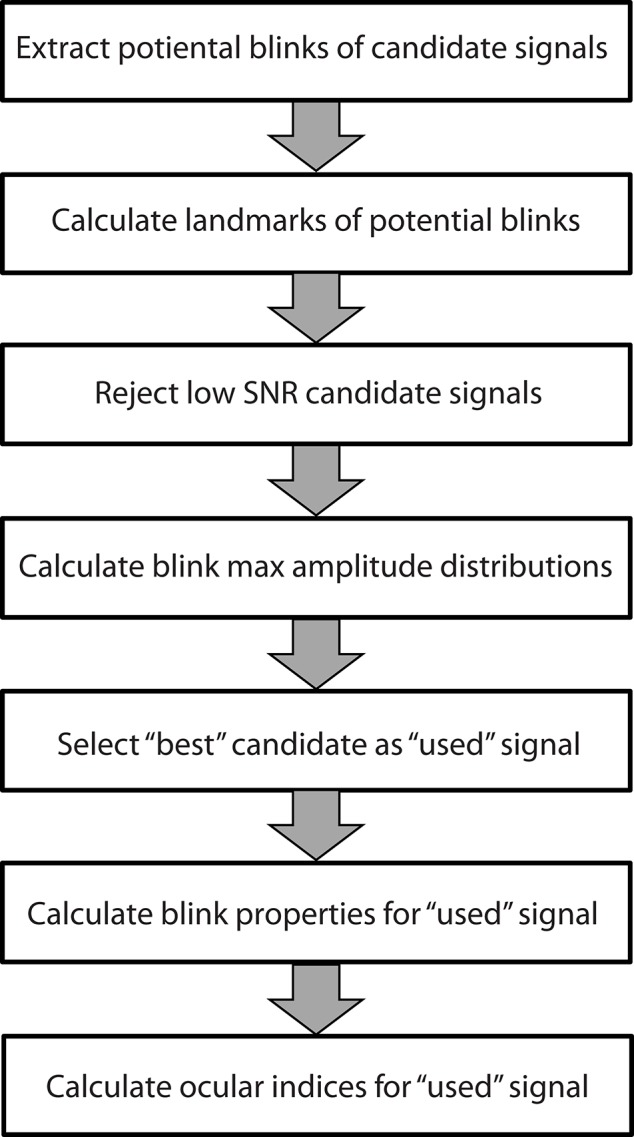
**Steps used by the BLINKER software to extract blink properties from time series**. Each step is described in a separate subsection of the Methods and materials.

The blink extraction algorithm takes an array of candidate time series and selects the best candidate signal from those provided. Figure 1 summarizes the steps of the algorithm. The following sections discuss these steps in more detail. BLINKER provides a number of post processing utilities to calculate various properties of individual blinks and to compare the positions of the blinks calculated from multiple signals or methods.

#### Preprocessing and potential blink detection

Regardless of the input signal type, BLINKER uses the same method for initial blink detection and preliminary calculation of blink start and end times. Each candidate signal is band-passed filtered in the interval [1, 20] Hz prior to blink detection. BLINKER then determines the intervals during which the signal is greater than 1.5 standard deviations above the overall signal mean. These intervals form the *potential blinks*. We consider only potential blinks that are longer than 50 ms and are at least 50 ms apart. These criteria eliminate many small rapid eye movements without appearing to eliminate many actual blinks.

#### Calculating blink landmarks

**Figure 2:**
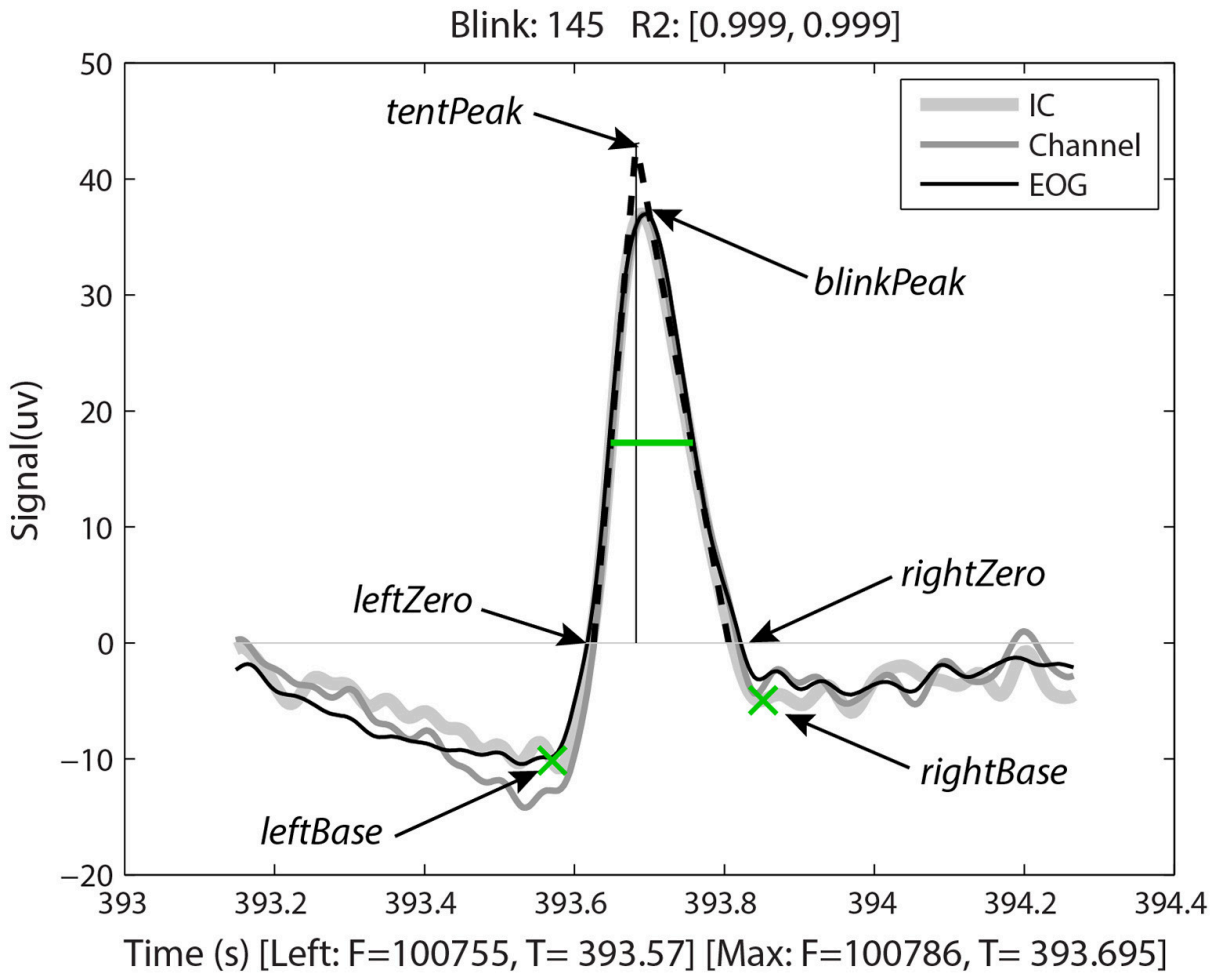
**Output of BLINKER software showing various blink landmarks**. The figure overlays three different time-series (an independent component, an EEG channel, and an upper vertical EOG channel, respectively) corresponding to the time of the blink. The green crosses mark the *leftBase* and *rightBase*, respectively. The green horizontal line marks the *half-zero duration*. The dotted black lines represent the best linear fits to the *upStroke* and *downStroke* of the blink, respectively. The thin vertical line is the perpendicular from the *tentPeak* to the zero line.

Having identified the potential blinks, BLINKER then applies a fitting process to find specified landmarks for each blink and saves information about each candidate blink in a structure. Figure 2, produced by BLINKER using its blink plot overlay function, illustrates the blink fitting process for a prototypical blink. In the example of Figure 2, BLINKER overlays signals corresponding to an independent component (IC), an EEG channel, and an EOG channel for the same blink. The plot overlay function accepts a cell array of input signals and uses the first signal to compute and display the blink landmarks. In the example plot, the maximum value in the interval occurs at frame 100786 (the *maxFrame*), which is 393.695 s from the beginning of the dataset. If two or more points in the interval achieve the maximum value, the *maxFrame* is the first.

After determining the *maxFrame*, BLINKER computes the remaining blink landmarks as follows. The *leftZero* is the last zero crossing before *maxFrame*. If the signal does not cross zero between this blink and the previous blink, *leftZero* is the frame of the lowest amplitude between the blinks. The *rightZero* frame has a similar definition. The *upStroke* is the interval between *leftZero* and *maxFrame*, and the *downStroke* is the interval between *maxFrame* and *rightZero*. The *leftBase* (frame 100755 at time 393.57 s in the example of Figure 2) is the first local minimum to the left of the maximum velocity frame in the *upStroke*. Similarly, the *rightBase* is the first local minimum to the right of the maximum velocity frame in the *downStroke*. BLINKER marks the *leftBase* and *rightBase* with green crosses.

Stereotypical blinks have a rounded tent-like shape and BLINKER uses a tent-fitting strategy for characterizing blinks similar to that proposed in Caffier et al. (2003) for characterizing blink shape. BLINKER computes the best linear fits for the inner 80% of the *upStroke* and *downStroke*, respectively, for each potential blink in a candidate signal. The quality (*R*^2^) of the correlation of these lines (denoted by *leftR2* and *rightR2*, respectively) with the actual blink trajectory is a measure of the closeness of the potential blink to a stereotypical blink. For the blink of Figure 2, the correlations to the *upStroke* and the *downStroke* are both 0.999. BLINKER computes the intersection of these fit two lines (the *tentPeak*) and displays the perpendicular line using a thin black line. The *tentPeak* is slightly forward of and above the maximum of the actual blink trajectory (the *blinkPeak*) in a stereotypical blink. The values *R*^2^, as well as the relative position of the *tentPeak* to the *blinkPeak*, provide simple tests of the how closely the blink resembles a stereotypical blink.

#### Rejecting candidate signals with low SNR

After determining the landmarks for all of the potential blinks in a candidate signal, BLINKER computes the *blink-amplitude ratio* (*BAR*) for the signal. The *blink-amplitude* ratio is the average amplitude of the signal between the blink *leftZero* and *rightZero* zero crossings divided by the average amplitude of the positive fraction of the signal “outside” the blink. (The blink excursion is always in the positive direction.) The left outer portion consists of the interval from the *rightZero* of the previous blink (or the beginning of the signal for the first blink) to the *leftZero* of this blink. The right outer portion consists of the interval from the *rightZero* of this blink to the *leftZero* of the next blink (or the end of the signal for the last blink). BLINKER rejects signals that have a *BAR* outside a specified range ([3, 50] by default). We have found empirically that signals with *BAR* values in the range [5, 20] usually capture blinks reasonably well. *BAR* is a measure of the signal-to-noise ratio (SNR) of the blink to the background in a candidate signal.

#### Distinguishing blinks from other eye movements

BLINKER uses a process of thresholding and elimination of outliers to select the best signal from the signal candidates for identifying blinks. Blinks are tent-shaped and have a high amplitude relative to the background signal. Unfortunately, small movements of the eyeballs as well as large artifactual spikes can confound blink detection. We have found empirically that eye movements tend to have smaller amplitudes than the “best” blinks, and large artifactual spikes tend to have larger amplitudes than the “best” blinks. We use these observations about blink amplitudes to exclude non-blink eye movements.

**Figure 3:**
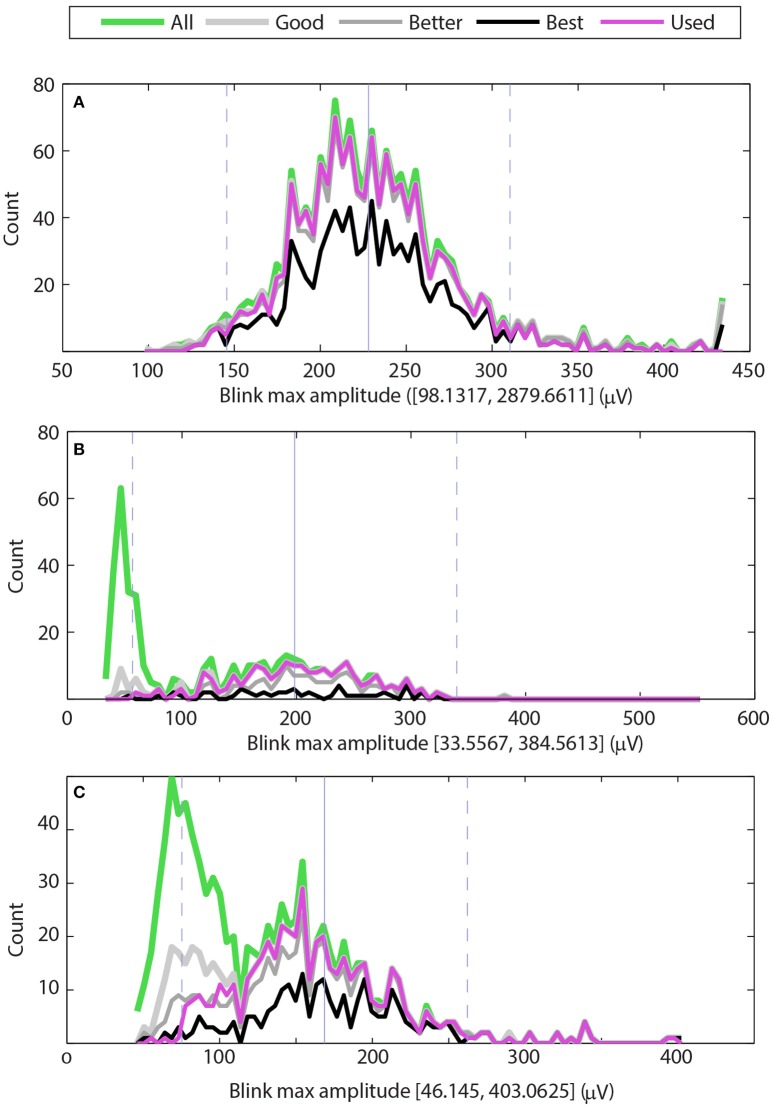
**Distributions of maximum blink amplitudes for the upper vertical EOG channel of three different datasets**. The green line shows the histogram for all potential blinks, and the thick light gray line shows the histogram for blinks whose *R*^2^ values are at least 0.90 (good). The medium gray line shows the histogram for blinks whose *R*^2^ values are at least 0.95 (better), and the black line shows the histogram of blinks with *R*^2^ values of at least 0.98 (best). The magenta line shows the histogram of blinks selected by BLINKER. **(A)** Dataset has a typical distribution of blinks with a few very large amplitude non-blink artifacts. **(B)** Dataset has a significant number of low-amplitude non-blink eye movements well-separated from normal blink amplitudes. **(C)** Dataset has a significant number of non-blink eye movements with moderate *R*^2^ values and amplitude distribution that overlaps with the normal blink amplitude distribution.

Figure 3 shows examples of the maximum amplitude distributions for three typical cases. The green line shows the distribution of maximum amplitudes of all potential blinks, and the thick light gray line shows the distribution of maximum amplitudes of the “good” blinks (*upStroke* and *downStroke R*^2^ ≥ 0.90). The medium thick gray line shows the distribution of maximum amplitudes of the “better” blinks (*upStroke* and *downStroke R*^2^ ≥ 0.95), and the black line shows the distribution of maximum amplitudes of the “best” blinks (*upStroke* and *downStroke R*^2^ ≥ 0.98). The magenta line shows the blinks selected by BLINKER. These “used” blinks have *upStroke* and *downStroke R*^2^ ≥ 0.90 and satisfy the *maximum amplitude distribution criterion* and the *pAVR criterion*. These later criteria allow BLINKER to separate normal blinks from eye movements.

The *maximum amplitude distribution criterion* enforces the bell-shaped maximum distribution that normal blinks have around the median of the “best” blinks. This criteria eliminates blinks whose *R*^2^ is low and whose amplitudes are far from the best blink median. By default, BLINKER eliminates “best blinks” more than five robust standard deviations from the median and “good” blinks more than two robust standard deviations away from this median. Here we define the robust standard deviation as 1.4826 times the median absolute deviation from the median. Figure 3 displays the median of the “best” blinks with a gray vertical line and the locations that are two robust standard deviations from this median with dashed gray lines.

The *pAVR criterion* captures the difference between the sharp rising edge of saccades and the more curved rise of normal blinks. We have found empirically that blink candidates with *pAVR* ≤ 3 do not correspond to normal blinks, but rather saccades having short, fast eye movements.

The three graphs in Figure 3 correspond to datasets from three different subjects in a driving study. In all three cases, BLINKER selected the EOG detector above the right eye as containing the best blinks. The maximum candidate blink amplitudes for the dataset of Figure 3A range from 144 to 2880 μ*V* with a median of 224. Most of the blinks in the two robust-standard-deviation range [139, 309] have *R*^2^ ≥ 0.90 (gray line). However, the dataset also has some large excursions corresponding to artifacts, as indicated by the upper end of the range of blinks amplitude maxima (2879.6611). The BLINKER maximum amplitude distribution criterion excludes these excursions.

Figure 3B shows a second type of typical distribution for candidate blink maxima. Here the candidate blink maxima fall into two, fairly well-separated groups. Video verification of the blinks corresponding to the large green peak on the left indicate that these are small amplitude eyeball movements and not blinks. Most of the blinks with *R*^2^ ≥ 0.90 fall in a bell-shaped distribution that is within two robust standard deviations from the median of the “best” blinks.

Figure 3C shows a dataset that also has two distinct distributions of candidate blink maxima. In this case, the two distributions are not well-separated, and many of the features in the left distribution have reasonably high *R*^2^ values. Video verification of the blinks corresponding to the large green peak on the left indicate many saccades and complex eye movements. Here the *pAVR criterion* eliminates many of these spurious motions.

#### Choosing the best signal from candidates

BLINKER requires a list of candidate EEG, EOG, or IC time series from which to select the best signal for determining the blinks. Frontal EEG channels and vertical upper EOG channels usually provide the best signals for capturing blinks. However, contamination by artifacts can sometimes render the “obvious” channel unusable. For EEG channels, we usually provide several frontal channels and allow the algorithm to detect the channel that best captures blinks. This approach allows the algorithm to compensate for noisy channels and poor connections. If the user provides a list of the EOG channels, the algorithm usually automatically selects the upper vertical EOG channel from among the set of channels. This feature is useful in case the channels have been mislabeled (which does happen in our experience) and as a cross check on blink quality. If the user doesn't specify which channels to use, BLINKER tests all available channels and quite successfully chooses the best channel.

To select the “best” signal from candidate signals, BLINKER first tests whether any candidate signals have at least 70% of the blinks within two-standard deviations of the median of the “best” blinks (good blink ratio criteria). From the set of candidates meeting this criterion, BLINKER selects the candidate signal with the most “good” blinks. If no candidate signals meet the good blink ratio criteria, BLINKER selects the signal with the most “good” blinks and designates the signal as a “marginal” signal. This criteria works equally well for EOG channels and for EEG channels, although EEG channels tend to have somewhat lower percentages of good blinks than the upper vertical EOG channel.

#### Selecting the “best” independent component

To use independent components instead of EEG or EOG channels, you must provide the ICA decomposition, and BLINKER automatically selects the “best” IC to use. BLINKER uses the same process as described in the previous subsection for channels with a separate initial step that uses EyeCatch (Bigdely-Shamlo et al., 2013) to isolate probable eye-related ICs. EyeCatch compares a given IC spatial distribution (IC scalp map interpolated on a 64 × 64 grid) to a database of typical eye-related scalp maps and provides a relative measure of how likely it is that a given IC contains eye-related artifacts. Typically, of the ICs identified as eye-related, only a few contain eye blink phenomena, with the rest containing various other eye-related activations. Eye-blink ICs have spatial distributions that are laterally symmetric, and significantly weighted to the front of the scalp map. To select these eye-blink ICs from the list of eye-related ICs provided by EyeCatch, BLINKER computes a figure-of-merit as the difference between the means of the frontal and rear hemisphere distributions of each component interpolated scalp map. BLINKER reorients the selected ICs so that the average spatial distribution of the frontal hemisphere is always greater than that of the rear hemisphere.

After selecting candidate “blink” ICs, BLINKER uses the procedures described in Section Preprocessing and potential blink detection, Calculating blink landmarks, Rejecting candidate signals with low SNR, Distinguishing blinks from other eye movements, and Choosing the best signal from candidates. The calculation of blinks using ICs requires a headset with sufficient detector density to get a reasonable estimate of the IC scalp maps. Using ICs instead of channels can improve blink detection for certain noisy datasets. However, IC-based blink detection can also be sensitive to minor shifts in headset position during the experiment. In this case, one IC component captures the blinks before the cap shift and another IC component captures the blinks after the shift. For this reason, we recommend starting with EEG or EOG channels for blink detection or using channels in combination with ICs. BLINKER supports merging of blinks detected from different methods with indications of how many methods produced a particular blink.

### Computation of ocular indices

The ocular indices that BLINKER computes include blink rate, blink duration, and blink amplitude-velocity ratios. BLINKER also computes several other measures as well as statistics characterizing the variability of these measures.

#### Blink rate

We define *blink rate* as the number of blinks per minute. Our blink detection algorithm always assigns a single maximum frame to each blink. We compute the average blink rate for the dataset as the number of blink maxima divided by the total length of the dataset in minutes. Users can easily detect instantaneous blink rates by computing the number of blinks in sliding window. Literature suggests that instantaneous blink rates be averaged over intervals of at least 3 to 5 min (Zaman and Doughty, 1997).

#### Blink duration

In eye tracking analyses, researchers often define blinks as eyelid movements in which the pupil is more than half occluded. We have used several different methods of calculating blink duration. The *half-zero duration* is the width of the blink in seconds at half of the blink amplitude from the zero level, as displayed by the green horizontal line in Figure 2. The low standard deviation (described below) suggests that the *half-zero duration* is the least sensitive to the effects of complex blinks and eye movements. Similarly, *half-base duration* is the width at half of the blink amplitude measured from the *leftBase* of the blink. The *half-base duration* is slightly longer than the *half-zero duration*, although these measures are highly correlated. The *base duration* is *rightBase*–*leftBase* and *zero duration* is *rightZero*–*leftZero*. These latter measures vary considerably for complex blinks or blinks contaminated by other eye movements (Caffier et al., 2003). The *tent duration* is the difference between the intersections of the *downStroke* and *upStroke* linear fit lines with the zero line.

#### Velocity measures

Johns (2003) has proposed two velocity measures as potential indicators of fatigue: The positive amplitude velocity ratio (*pAVR*) and the negative amplitude velocity ratio (*nAVR*). The positive amplitude velocity ratio is the ratio of the maximum amplitude of the blink over the maximum velocity (rate of change) during the blink *upStroke*. Similarly, the negative amplitude velocity ratio is the ratio of the maximum amplitude of the blink over the maximum velocity found in the blink *downStroke*. These measures have units of *centiseconds*.

## Results

We computed the blink statistics for the data collections described in Table 1. We analyzed ~675,000 putative blinks extracted from ~590 h of EEG recordings, allowing BLINKER to select the best channel. We used raw data for the calculations as described in Section Data preprocessing. Both ARL-Shoot and BCI2000 consisted of multiple tasks for a given subject recorded in a single session, and the individual task files for these collections were too short to get good estimates of the blink maxima distributions. We used the combined datasets to determine the blink distributions for channel selection for these two collections.

**Figure 4:**
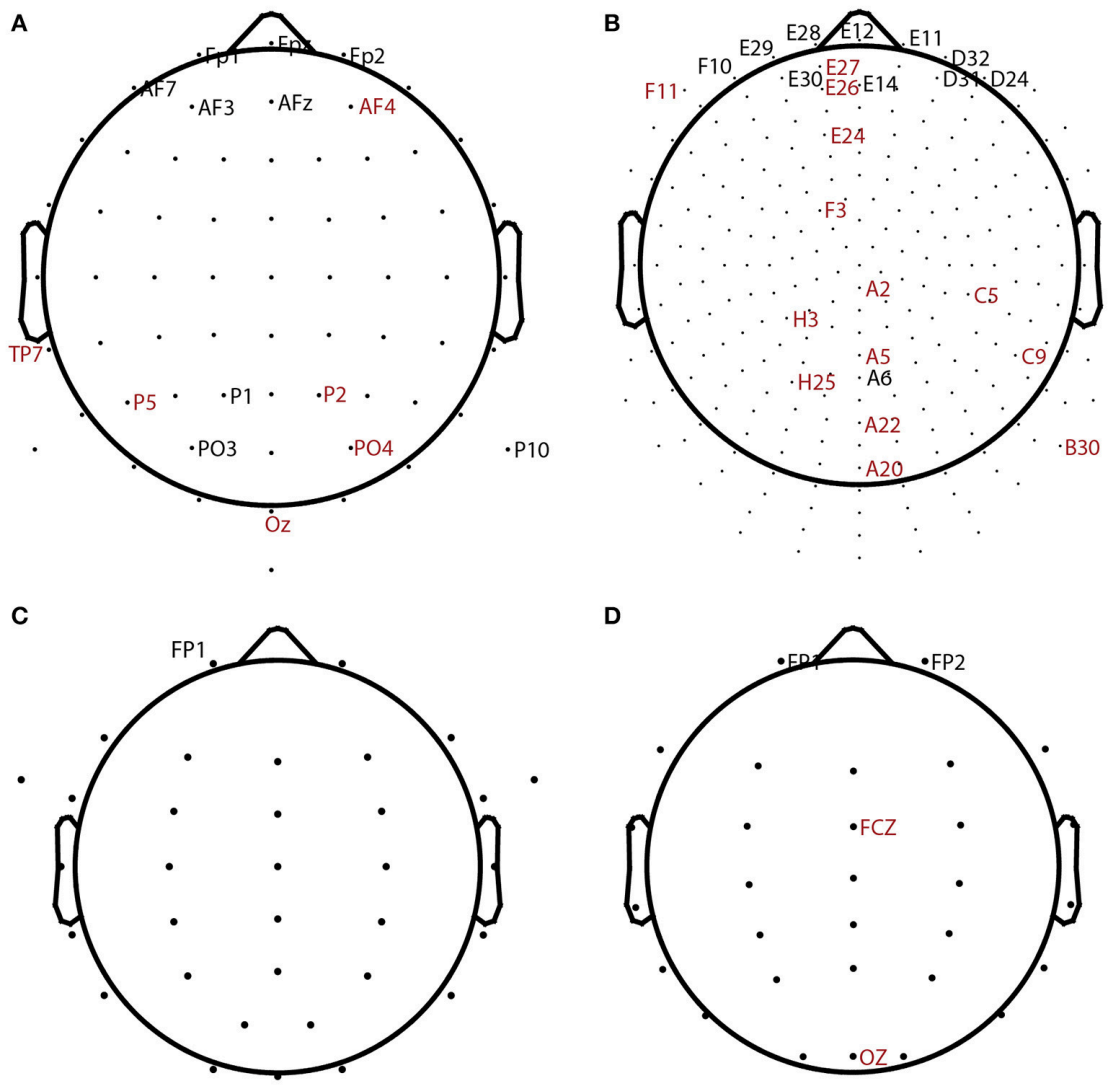
**BLINKER channel selection for different datasets**. Dots show positions of candidate channels. Labeled channels were selected. Channels labeled in red were picked for only 1 dataset. **(A)** 64-channel ARL-BCIT driving collection (also has 4 EOG channels). **(B)** 256-channel ARL-BCIT driving collection (also has 4 EOG channels). **(C)** 34-channel ARL-Shoot data (also has 4 EOG channels). **(D)** 32-channel NCTU-LK driving data collection.

Table 2 summarizes the overall statistics and the results of automatic channel selection. The ARL-BCIT data collection contains two types of headsets (64 channel and 256 channel EEG) as illustrated in Figures 4A,B, respectively. Figure 4 indicates the channel positions for the headsets using dots. In addition each BCIT dataset includes 4 additional EOG channels placed vertically above the right eye (*veou*), vertically below the right eye (*veol*), horizontally on the outside of the right eye (*heor*), and horizontally on the outside of the left eye (*heol*). BLINKER was able to identify blinks in 662 out of the 669 ARL-BCIT datasets. In 541 of these datasets, BLINKER selected the vertical upper EOG channel (*veou*) as having the best blink signal. Channels selected by BLINKER as the best in at least one dataset are labeled in Figure 4, with channels selected by only one dataset labeled in red. For the most part, BLINKER automatically picks a frontal channel when the vertical EOG channel is not suitable.

**Table 2:** **Percentage of datasets for which BLINKER succeeded in computing blinks using channels/EOG as well as the overall average total blinks per minute for each collection and the total number of blinks**.

**Collection**	**Successful (%)**	**Marginal (%)**	**Selected channel for all datasets**	**Mean rate**	**Total blinks**
ARL-BCIT	620 (93%)	44 (7%)	veou(291), fpz(13), fp1(8), p1(6), af7(6), po3(5), fp2(5), veol(3), afz(3), p10(2), af3(2), tp7(1), po4(1), p5(1), p2(1), oz(1), af4(1),	19.6	514K
			veou(250), e12(10), e29(7), e28(5), e11(5), f10(4), d32(3), d31(3), d24(3), e30(2), a6(2), e14(2), h3(1), h25(1), f3(1), f11(1), e27(1), e26(1), e24(1), c9(1), c5(1), b30(1), a5(1), a22(1), a20(1), a2(1)		
ARL-Shoot	99 (79%)	27 (21%)	veou(117), fp1(9)	22.4	19.5K
NCTU-LK	53 (66%)	27 (34%)	fp1(53), fp2(25), fcz(1), oz(1)	21.4	137K
BCI-2000	961 (73%)	109 (8%)	fpz(253), fp1(146), f8(135), afz(103), af4(83), fp2(81), af8(48), af7(45), f7(39), f4(36), f6(30), ft8(24), af3(24), f2(8), f1(7), p1(3), t9(2), tp7(1), fz(1), fc6(1)	22.6	60K

*ARL-BCIT selected channels are separated by headset type (64 vs. 256 channels)*.

Figures 4C,D show the channel layouts for ARL-Shoot and NCTU-LK, respectively. The ARL-Shoot data collection also includes 4 EOG channels in the same positions as those defined above for ARL-BCIT. Again, BLINKER usually chooses the vertical EOG channel when it is available or one of the frontal channels when it is not suitable or not available.

The BCI-2000 data collection uses a 64-channel headset, and BLINKER for the most part selects frontal channels. This subject's data consists of 14 relatively short tasks, recorded consecutively in the same session. As with the ARL-Shoot data, we combine data across each session to get a better estimate of the blink maximum amplitude distributions for final channel selection. Three subjects had poor quality signals, and BLINKER failed to detect blinks for several of the tasks within those subject's sessions. The tasks for which BLINKER returned blinks in these subjects were not frontal channels and are shaded in gray in Table 2. Selection of appropriate channels is an indicator of blink detection quality as discussed further below.

### Analysis of blink rate

Average blink rates over the collection ranged from 19.6 to 22.6 blinks/min, well within the range of values reported in the literature (Cruz et al., 2011) for spontaneous blink rates. Table 2 also indicates the channels BLINKER selected as the optimal “blink” channel. ARL-BCIT included a mix of 64 channel and 256 channel headsets; hence, the two lists of channels. BLINKER often selected the front center channel, but sometimes channels slightly back had stronger signals or fewer non-blink artifacts. EEG channels in general have a smaller ratio of “good” blinks to potential blinks than the vertical upper EOG channel. This is expected, as EEG channels are positioned at a greater distance from the eyes and should have other content besides blinks.

Datasets in ARL-BCIT and ARL-Shoot each contained four EOG channels: VEOU (above right eye), VEOL (below right eye), HEOR (to right of right eye), and HEOL (to the left of left eye). We always ran BLINKER with all four channels. Usually, BLINKER automatically selected VEOU as the optimal blink channel. We manually examined the datasets for which BLINKER did not select VEOU. Several of these had VEOU channels with weak signal quality or large artifacts. Usually the values of blink parameters such as the blink amplitude ratios for these datasets were close to the border for failed blink detection. Thus, we think it is useful to present the algorithm with all EOG channels as a cross check and to examine manually datasets where BLINKER does not select the expected signal.

Separately from the other tasks, the ARL-Shoot study ran an “eyes closed” task of ~3-min duration. As a test of the algorithm, we also ran the blink detector on these datasets for all 14 subjects. All 14 of the eyes closed datasets failed completely as expected.

### Blink duration

We can also calculate blink duration using several different landmarks as described in Section Calculating blink landmarks. Table 3 presents a summary of the mean (std) dataset blink duration using different landmarks for computing duration. As expected, the *half-zero duration* method has the lowest standard deviation and a mean that is most consistent with reported values. As discussed in Section The BLINKER software, the *half-zero duration* corresponds closely to measuring the duration from when the eyelid first crosses the pupil. Eye trackers that rely on pupil detection also generally use this point as the starting point of the blink. The standard deviation of the duration for prototypical (good) blinks is quite small, as expected, based on the criteria for determining “good blinks.” The average *half-zero duration* for the good datasets (good blink to potential blink ratio > 0.70) is very close to the average *half-zero duration* computed on all datasets. Further, the average *half-zero duration* from EEG and EOG channels is also in good agreement. The NCTU-LK collection has blink durations that are somewhat longer and more variable than the other collections. This is consistent with the experimental paradigms. The NCTU-LK experiment was designed to measure fatigue in simulated night-time driving, and each experiment ran an hour or more.

**Table 3:** **Mean and standard deviation of blink duration in seconds using different methods**.

**Collection**	**Half-zero**	**Half-zero good data**	**Half-base**	**Zero**	**Base**	**Tent**
ARL-BCIT	0.12 (0.05)	0.12 (0.05)	0.14 (0.10)	0.25 (0.09)	0.32 (0.10)	0.23 (0.10)
ARL-Shoot^*^	0.12 (0.05)	0.12 (0.05)	0.14 (0.08)	0.24 (0.08)	0.29 (0.09)	0.22 (0.09)
NCTU-LK	0.14 (0.06)	0.13 (0.06)	0.16 (0.09)	0.28 (0.11)	0.34 (0.11)	0.27 (0.12)
BCI-2000^*^	0.11 (0.04)	0.11 (0.04)	0.12 (0.06)	0.24 (0.07)	0.32 (0.08)	0.21 (0.07)

*Datasets marked with asterisks indicate that signal selection used session data*.

**Figure 5:**
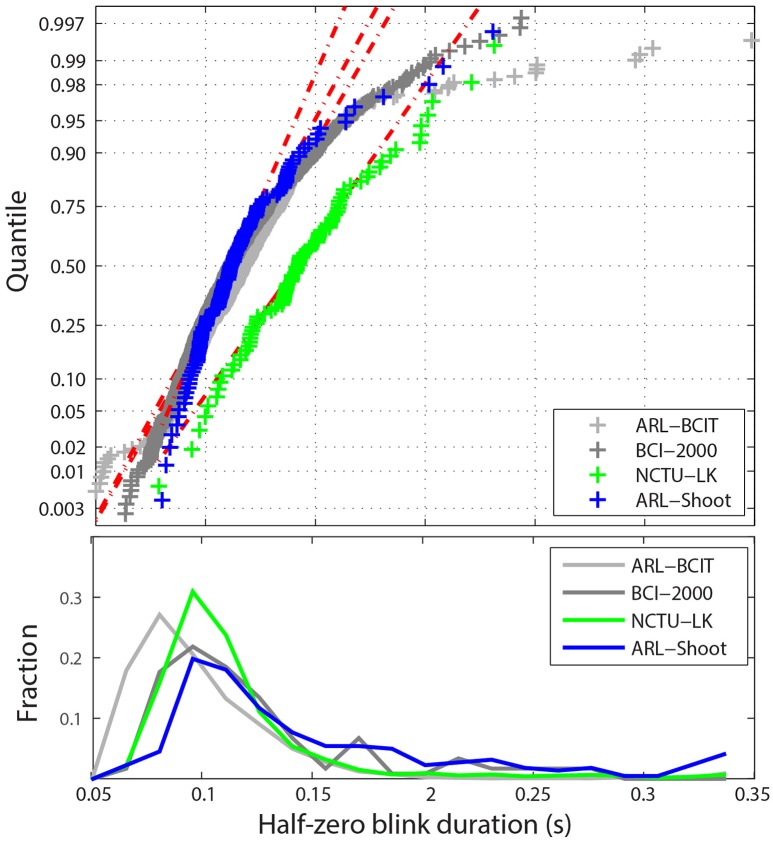
**Distribution of blink durations for the four collections**. Top panel shows a quantile plot against the normal distributions of the average half-zero blink duration for each dataset. The red dotted lines represent the best-fit normal distributions for the each of the four collections. Bottom panel shows the histogram of individual half-zero blink durations for each collection.

Figure 5 shows the statistical distribution of the *half-zero durations*. The upper graph shows a MATLAB normal probability plot of the mean blink durations for each dataset in the four collections. The horizontal axis displays the dataset average values in the range [0.05, 0.35] s. The vertical axis represents the corresponding quantile if the data were normally distributed. The vertical tic marks represent various standard quantiles. The red dotted lines correspond to the best-fit normal distribution. While most datasets fall well along a normal distribution, there are some datasets with very long average blink durations due mostly to some unusually long blink durations. Many of these longer blinks include more complex eye movements or are fatigue related. For example, the NCTU-LK study, which measures the effects of fatigue on driver response, includes some subjects who appear to drowse off for short periods. The lower panel of Figure 5 displays the fraction of half-zero blink durations in different intervals from 0.05 up to 0.35 s. This graph represents the 675K individual blinks in the four data collections. The horizontal axis is truncated at 0.35 s.

Figure 5 uses raw data. However, as mentioned above, ARL-Shoot and NCTU-LK are mastoid-referenced. Referencing makes little difference in blink detection, but can slightly shift the values of ocular indices such as blink duration. The referencing status of BCI-2000 is unknown. Durations computed for referenced data may shift slightly in either direction since the position of the zero mark relative to the blink may shift. Our computations of durations for referenced and non-referenced data did not vary significantly from each other on average.

### Ocular indices and tasks

In this section, we demonstrate how analysis of blinks can discover patterns and validate observations on a large scale. We focus on the ARL-Shoot dataset, which has 14 subjects and 9 different tasks. Four of these tasks (SEO2, SEO4, SEF2, SEF4) were shooting only (indicated by S in their label). Their targets were only enemies (indicated by EO in the label) or both enemies and friends (indicated by EF). The numerical notations in the labels (2 and 4) distinguish how many seconds the target remains visible. The study also includes dual tasks (indicated by Ds in their labels) which consisted of simultaneous shooting and performing arithmetic (DEO2, DEO4, DEF2, DEF4). There is also an arithmetic only (ARIT) task in the study.

**Figure 6:**
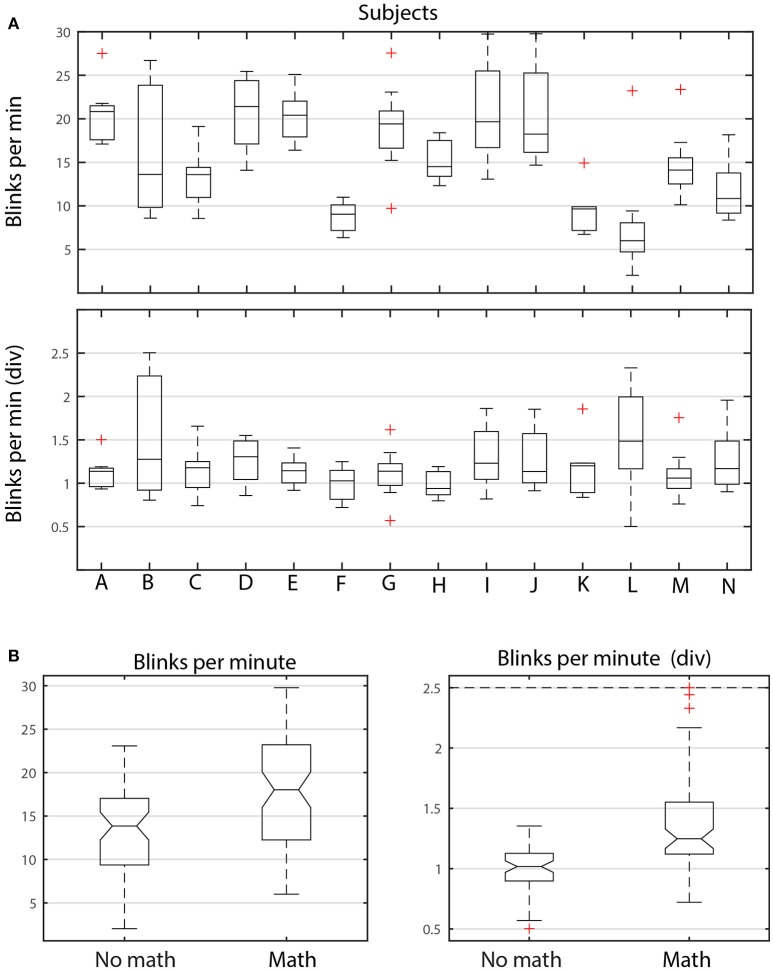
**ANOVA analysis of blink rate for ARL-Shoot. (A)** Top panel shows the distribution of average dataset blink rate for each of the 14 subjects (A through N) over the 9 tasks. The bottom panel shows the same distributions when each average is divided by the average of the dataset blink rates for no-math tasks. **(B)** Left panel compares the distributions of dataset average blink rates, grouping the math and no-math tasks for all subjects. The right panel shows the same distributions after dividing the dataset average blink rates by the subject's average no-math blink rate.

Figure 6 shows the distributions of average dataset blink rate in blinks/min for different subjects and different tasks. Figure 6A breaks down these dataset averages by subject. Each boxplot in the upper panel represents the distribution of the average dataset blink rate for the 9 datasets (corresponding to the 9 tasks) associated with each of the 14 subjects (A–N). The graph illustrates the considerable variability in average blink rate for the different subjects. Some subjects, such as K, have similar average blink rates among the tasks, while others subjects, such as B, I, and J, have considerable variability. Notice however, that there are subjects with both high medians and high variability (I and J). There are also subjects with low medians and high variability (B). Each subject appears to have intrinsic blink rate characteristics. However, within each subject, tasks involving arithmetic generally have higher blink rates than the shooting only tasks as can be seen by the left panel of Figure 6B. The median of the average dataset blink rate for no math tasks is significantly lower than the median of the average dataset blink rate for the tasks including math.

To better understand the interplay between inter subject and inter task variability, we scaled each subject's average blink rates by dividing by the average of the blink rates of the shooting only tasks (bottom panel of Figure 6A and right panel of Figure 6B, respectively). As shown by Figure 6B, the scaled median of the average shooting-only blink rates is significantly lower than the average of tasks involving arithmetic. We also found that average blink durations and *nAVR* were larger for tasks involving arithmetic than those only involving shooting. All of these indicators suggest that subjects spend longer time with eyes closed when doing arithmetic.

To quantify this relationship further, we performed an analysis of variance using the MATLAB *anovan* function. Table 4 shows the results of several variations on the analysis. The dataset has 14 subjects performing 9 tasks: 4 tasks consisting of shooting only, while the remaining 5 tasks include arithmetic with and without shooting. All of the analyses use *Subjects* and *Tasks* as the variables. The column labeled *Individual* shows the results of analysis using 14 levels for the *Subjects* variable and 9 levels for the *Tasks* variable. Because of the large variability in the base level of the individual subjects, all appear to be significantly different and the analysis does not reveal relationships. Clustering the tasks into *Math* and *No math* groups (designated *Grouped* in Table 4) reduces, but does not eliminate inter-subject variability.

**Table 4:** **Results of analysis of variance with subjects and tasks for the ARL-Shoot data collection**.

**Indicator**	**Variable**	**Individual**	**Grouped**	**Grouped with divided scaling**
Blink rate	Subject	*F*(13, 112) = 19.3, *p* = 3.7E−22	*F*(13, 112) = 15.9, *p* = 7.8E−20	*F*(13, 112) = 2.3, *p* = 0.01
	Task	*F*(8, 117) = 11.2, *p* = 2.3E−11	*F*(1, 124) = 49.0, *p* = 2.2E−10	*F*(1, 124) = 25.0, *p* = 2.2E−6
Blink duration	Subject	*F*(13, 112) = 13.4, *p* = 5.8E−17	*F*(13, 112) = 8.0, *p* = 4.0E−11	*F*(13, 112) = 1.1, *p* = 0.36
	Task	*F*(8, 117) = 11.7, *p* = 9.8E−12	*F*(1, 124) = 7.0, *p* = 0.0095	*F*(1, 124) = 8.4, *p* = 0.0046
*pAVR*	Subject	F(13, 112) = 22.0, *p* = 3.8E−24	*F*(13, 112) = 16.1, *p* = 5.2E−20	*F*(13, 112) = 0.88, *p* = 0.57
	Task	*F*(8, 117) = 6.1, *p* = 2.0E−6	*F*(1, 124) = 1.1, *p* = 0.29	*F*(1, 124) = 1.2, *p* = 0.28
*nAVR*	Subject	*F*(13, 112) = 36.0, *p* = 1.4E−32	*F*(13, 112) = 21.0, *p* = 3.4E−24	*F*(13, 112) = 1.5, *p* = 0.13
	Task	*F*(8, 117) = 12.6, *p* = 1.8E−12	*F*(1, 124) = 8.5, *p* = 0.0043	*F*(1, 124) = 9.3, *p* = 0.0028

The final column of Table 4 uses *div* data. Here we factor out each subject's intrinsic level by dividing a subject's values by the average of that subject's values on the non-math tasks. Several other scaling methods also worked for most indicators, although the division scaling method appeared to give the best results overall. As we can see from the last column of Table 4, with division scaling, the indicators show a significant difference between *Math* and *No math* categories, but little or no significant difference among the subjects. As indicated above, the values indicate a tendency to spend more time in the blinking state (i.e., higher blink rate, longer blinks, as well as slower up and down velocities) in the tasks involving arithmetic. As pointed out by Recarte et al. (2008) the relationship between blink rate and task can be quite complicated. They argue that mental workload of cognitive tasks increases blink rate while visual demand inhibits it. The results of our analysis points to the importance of factoring out subject effects in analyzing ocular indicators (Nezlek, 2007).

## The BLINKER software

BLINKER is freely available as a MATLAB toolbox at https://github.com/VisLab/EEG-Blinks. The toolset depends on EEGLAB (Delorme and Makeig, 2004) and can be run as an EEGLAB plugin. Researchers wishing to calculate blinks from independent components can also download EYECatch to restrict the selection to known eye components (https://github.com/bigdelys/eye-catch). This step is not necessary, but speeds up the computations when using independent components.

The simplest command to extract the blinks (blinks), their shapes (blinkFits), and their properties (blinkProperties) using BLINKER and the default settings is:

$$\begin{array}{l} {}[\text{EEG},\text{ com},\text{ blinks},\text{ blinkFits},...\\ \text{ blinkProperties},\text{params}]\;=...\\ \text{ pop}\_\text{blinks}(\text{EEG}) \end{array}$$

Here EEG is a structure that is compatible with the EEGLAB EEG structure. However, BLINKER only requires that the input data structure have an srate field containing the sampling rate in Hz and a data field containing an *m* × *n* array of *m* candidate time series, each consisting of *n* consecutive data frames sampled at srate Hz. The time series can be EEG channels, EOG channels, or ICs or combinations of these. This version brings up a GUI that allows users to set various paths and options. Alternatively, pop_blinks can take a second optional argument, params, a structure holding values that override default values. This latter version can be called from a script without a GUI for large scale automated processing.

The toolbox comes with many code examples of how to call the functions and the user is referred to the online user manual at http://vislab.github.io/EEG-Blinks/ for additional information.

## Visual verification of BLINKER output

In order to determine the correspondence between computed BLINKER landmarks and visual manifestations of blinks, we compared the output of the BLINKER blink detection algorithm to manual annotation of simultaneous video for several datasets. Video recordings of subjects' eyes were available for a number of ARL-BCIT datasets. We selected a subset of recordings based primarily based on how easy it was to determine eyelid position from the video recording. We eliminated datasets in which the subject wore glasses, was not looking directly at the camera, or had other physical features that made it difficult to score blinks.

We dumped the video, initially recorded at 250 fps, as individual images sampled at a rate of 60 frames per second using Adobe Premiere 6.0. Estimates of the synchronization of the start of the video and the start of the EEG were accurate to about 1 s. Further, the video frame rate was not perfectly uniform. Thus, we manually synchronized blocks of video with the EEG when we observed a drift. We then extracted the following data for the observed blinks:

- Frame of first eyelid movement
- Frame where eyelid is at top of pupil moving downward
- Last frame prior to eyelid reversal
- Frame where eyelid reaches the top of pupil moving upward
- Frame of last eyelid movement

**Figure 7:**
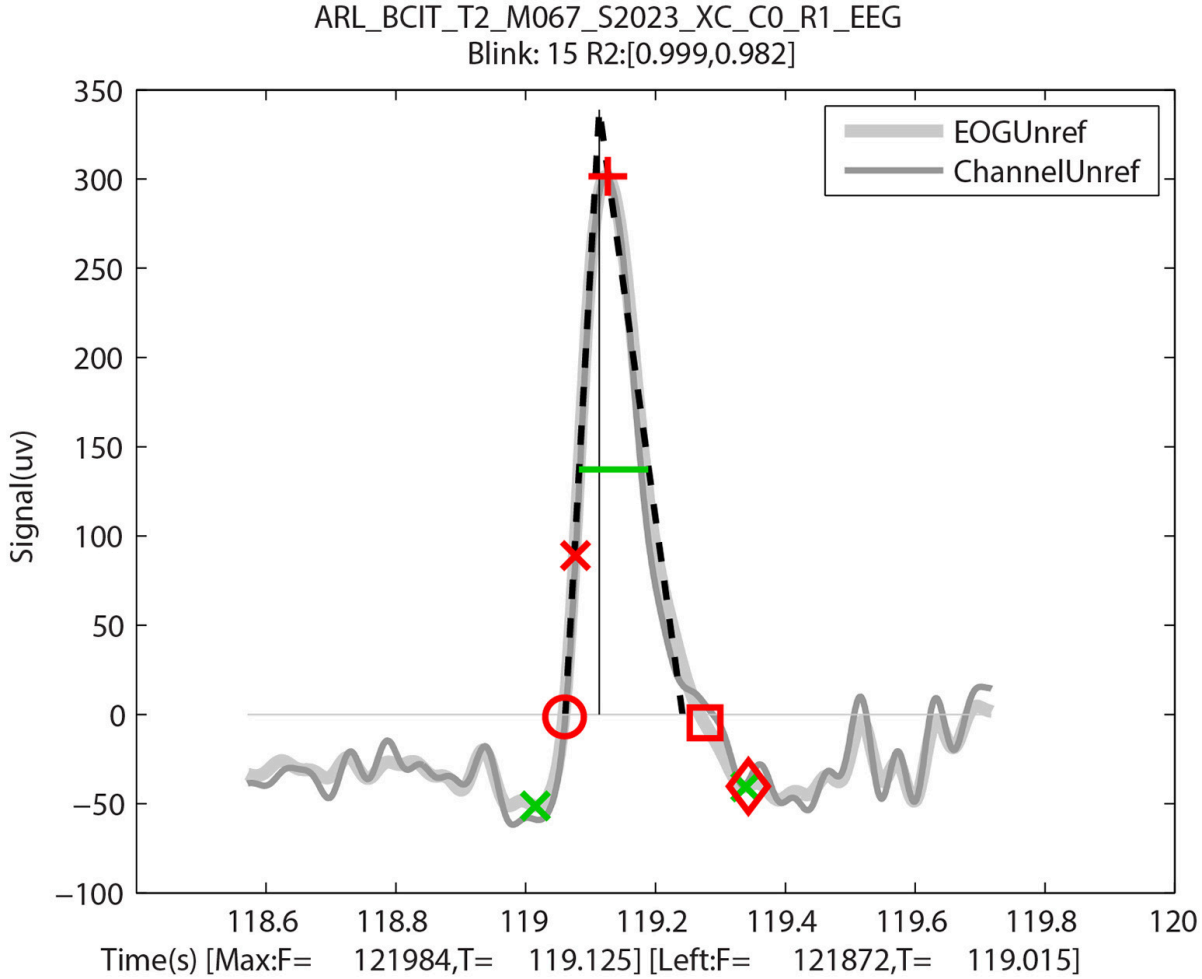
**An output by BLINKER of blink 15 of a specific ARL-BCIT dataset over-plotted with landmarks manually extracted from video: First eyelid movement (red circle), eyelid at top of pupil moving downward (red x), at eyelid reversal (red cross), eyelid at top of pupil moving upward (red square), and last detectable eyelid movement (red diamond)**. The x-axis label shows the frames and times in seconds of the *leftBase* point and point of blink maximum amplitude, respectively.

Although there was some variation from blink to blink, typically this variation was attributable to time resolution of the video. Figure 7 uses red symbols to mark a blink with these manually collected video landmarks plotted on the BLINKER output for the same blink. The first eyelid movement (red circle) is very close to the *leftZero* crossing, and the initial pupil crossing (red x) is close to the *half-zero* amplitude frame. The last frame prior to eyelid reversal (red cross) is close to the maximum frame. The pupil crossing during eyelid opening (red square) is close to the *rightZero* crossing, and the last eyelid movement (red diamond) is close to the *rightBase*. The green markers in this figure are from BLINKER and described in Figure 1.

To get a measure of detection accuracy and false positive rate, we compared the blinks detected by BLINKER to the video taken for various subjects. Some examples of representative datasets are shown in Table 5.

**Table 5:** **Video verification of correspondence between BLINKER detected blinks and visual manifestations of blinks**.

**Dataset**	**Figures**	**TP**	**FP**	**FN**	**Explanations**
BCIT S2007 X2	Figure 3A	143	0	10	Four were first of a blink pair with saccade between
					Four had a saccade at the beginning
					Two had a saccade at the end
BCIT S2008 XB	Figure 3B	32	0	6	Four had a saccade at the end
					Two were a pair of low amplitude blinks less than 0.1 s apart
BCIT S2018 XB	Figure 3C	17	0	1	Miss (FN) had amplitude slightly larger than high cutoff—obviously a blink from the trace.
BCIT S2019 X2	Similar to Figure 3B	25	1	1	False positive was a “blink-like” saccade
					Miss had a long tail

*TP, true positives; FP, false positive (BLINKER yes, video no); FN, false negatives (BLINKER no, video yes)*.

The first dataset of Table 5 has the maximum blink distribution shown in Figure 3A. This dataset is very clean. This dataset had no false positives, but the algorithm missed 10 out of the 143 blinks using the default settings. Examination of the 10 misses showed that four of them were members of a blink pair less than 0.5 s apart with one blink in the pair being detected and the other missed. All of the missed blinks either had long tails or were at the beginning or end of a saccade, which usually elevated the signal so that it did not fall below zero on one end of the blink or the other. The second dataset of Table 5 had many candidate blinks that were low amplitude eye movements (Figure 3B), which BLINKER successfully eliminated. BLINKER missed six of 38 blinks—four of which were confounded with saccades. Although the third dataset had a very difficult maximum amplitude distribution (Figure 3C), BLINKER successfully eliminated all eye movements in the examined segment. The one miss was clearly a blink, but its amplitude was greater than five robust standard deviations above the best blink median, and so BLINKER eliminated it. The final dataset had one miss and one false positive, both involving saccades.

BLINKER provides a method of grouping candidate blinks from multiple channels and displaying the individual candidates in a convenient way. BLINKER forms blink groups when at least one usable signal goes above 1.5 robust standard deviations above zero. BLINKER overlays all of the usable signals, displaying the blink candidates in this group with solid lines and non-members of the group with dashed lines.

**Figure 8:**
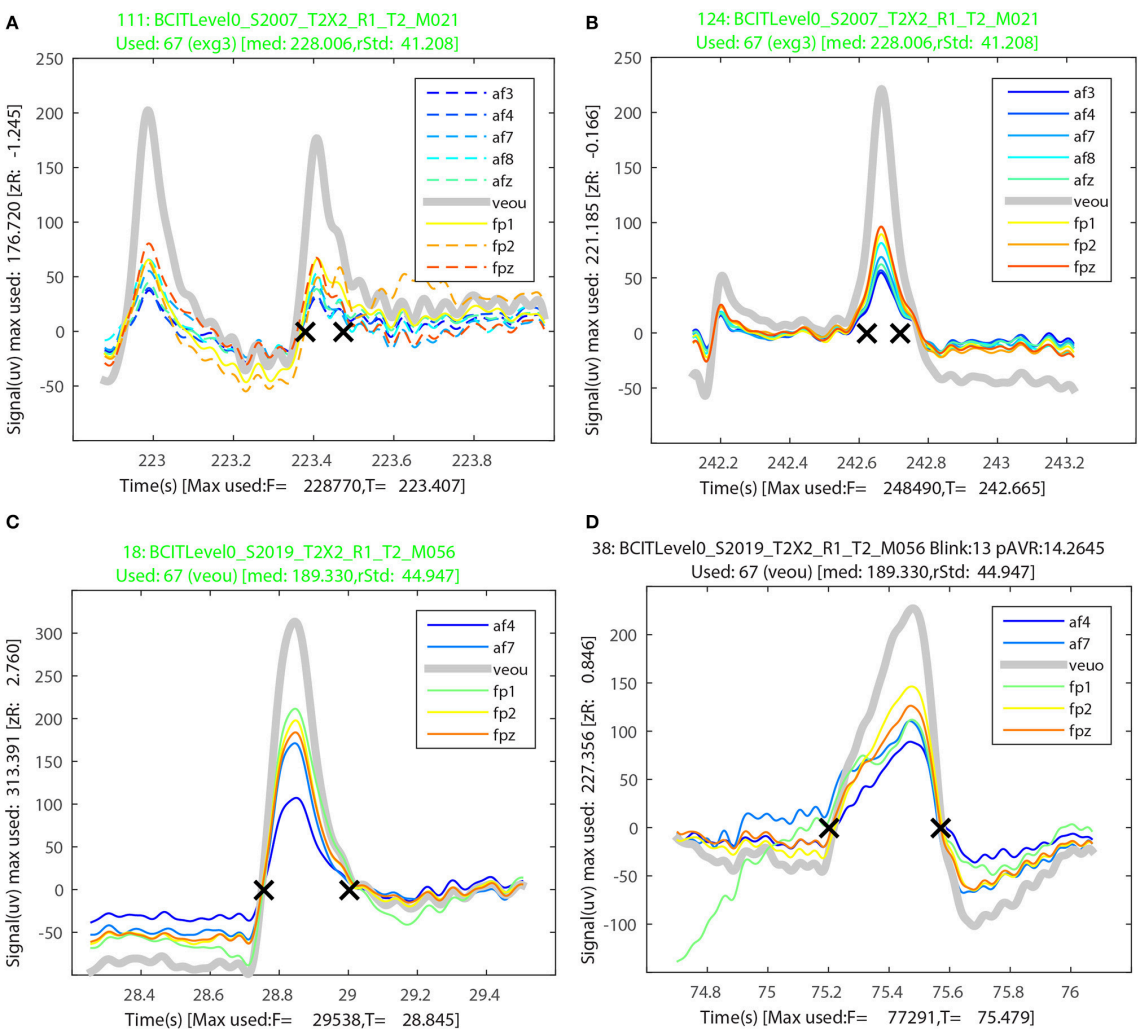
**Sample output from the BLINKER group display for the first and last datasets from Table **5**. (A)** A miss with two closely spaced candidates intermixed with a saccade. **(B)** A miss with a leading saccade. **(C)** A miss with a leading saccade. **(D)** A falsely detected blink that was a saccade.

Figures 8A,B illustrate the BLINKER display of blink groups for two misses of the first dataset in Table 5. Figures 8C,D illustrate the BLINKER display for the miss and the hit of the last dataset in Table 5. The runs of Table 5 were made by providing all 64 EEG channels plus 4 EEG channels as potential signals and allowing BLINKER to select the usable signals. For the first dataset, BLINKER designated eight of the EEG channels and the upper vertical EOG channel as “usable” and selected the vertical EOG channel (*veou*) as the best choice. Figure 8A shows a blink that closely follows another (correctly detected) blink. Only *veou* has this as a blink candidate as the signal has too low an amplitude in the other channels to be noticed. Further, this miss has a long tail and a close examination indicates that a horizontal eye movement confounded the detection. Figure 8B is also from the first dataset. This blink appears as a candidate in all of the usable signals, but here an eye movement preceding the blink confounds detection. The titles for both of these figures appear in green because the used channel (*veou*) has the blinks as a blink candidate. When the used channel is not a member of the blink group, the title appears in red. A black title indicates that the blink candidate is an actual blink with respect to the used signal.

Figures 8C,D show two examples from the last dataset of Table 5. The blink candidate of Figure 8C had a saccade directly before the start of the blink. The blink candidate of Figure 8D was detected as an actual blink, but appears to have actually been a saccade.

## Discussion

Psychologists have long understood that pupillary responses and other ocular indices contain information about emotion and other characteristics of subjects (Mathôt and der Stigchel, 2015). Opthmologists have measured blink rates and other ocular properties to assess disease states such as glaucoma (Cruz et al., 2011; Doughty, 2014). Often researchers have computed these indices manually from video. More recently however, special devices such as a contact lens sensors (CLS) have been developed to extract blinking activity and other eye properties under continuous monitoring (Gisler et al., 2015). Hsieh and Tai (2013) proposed the design of a dedicated electronic sensing pad affixed on the forehead above the eye to measure blink durations. Very little work has been done to study eye-blinking activity extracted from EEG. Plöchl et al. (2012) have done a comprehensive study of how different types of eye artifacts manifest in EEG. They decompose EEG using ICA and then identify components that capture eye artifacts based on relationships of these components to eye tracker information. They have also provided a MATLAB toolbox called EYE-EEG that allows users to analyze eye-tracking data in conjunction with EEG. Other tools, such as EALab (Andreu-Perez et al., 2015) calculate ocular indices from eye-trackers independently of EEG. EALab focuses on pupillometry measures as well as saccades and fixations.

Our motivation for developing BLINKER is that much information about blinking and eye movements is available “for free” in EEG and can be extracted automatically for long stretches of EEG data. As far as the authors are aware, BLINKER is the first tool designed to extract ocular indices from EEG data in a standardized way. BLINKER makes it possible to post-process virtually any previously acquired EEG dataset to extract the behavioral information embodied in several common ocular indices. BLINKER provides an opportunity to gather blink statistics and information about blink variability over a much larger and diverse subject pool than has been previously available. Our collaborators have also used BLINKER for mundane purposes such as extracting and/or verifying sync markers for EEG and eye tracking.

Our manual video verification shows that the BLINKER algorithms are effective in capturing a majority of the blinks. However, subjects are highly variable in their behavior, which can contaminate the EEG signal in a variety of ways. Eye irritation, scratching, rubbing, squinting, and alternate rapid winking behaviors observed in video are reflected in atypical EEG signals. Saccades present a difficult detection problem, so tasks that involve extensive visual searching across a large visual field will be difficult for BLINKER. However, we believe that over long recordings, the effects of these difficulties will be minimal.

Our analysis of a large EEG collection has revealed a variety of spontaneous blinking behaviors that BLINKER can detect from EEG. We have also demonstrated how to isolate ocular index dependence on task. Recently, Wascher et al. (2015) have argued that blinks play a fundamental role in human information processing. The ability of BLINKER to accurately time individual blinks to stimuli and neural activity in EEG may provide a valuable investigative tool to researchers. BLINKER is freely available at https://github.com/VisLab/EEG-Blinks.

## Ethics statement

This study is exempt in that it is the reanalysis of de-identified data. The original data for these studies were approved under human subjects boards at their respective institutions.

## Author contributions

KK performed much of the initial exploratory algorithm development, participated in the development and testing of the algorithms and assisted in writing the paper. NB helped in exploratory algorithm development and participated in the writing of the paper. SK provided data for testing, assisted in the interpretation of results, and assisted in the writing of the paper. KR conceived the study, participated in the development and testing of the algorithms, wrote the software documentation, and led the writing of the paper.

### Conflict of interest statement

The authors declare that the research was conducted in the absence of any commercial or financial relationships that could be construed as a potential conflict of interest.
